# 18F-NaF PET/CT imaging of bone formation induced by bioactive glass S53P4 after mastoid obliteration

**DOI:** 10.1186/s41824-019-0065-3

**Published:** 2019-10-31

**Authors:** Adriana J. Timmermans, Jasper J. Quak, Petronella J. Hagen, David R. Colnot

**Affiliations:** 1 Department of Otorhinolaryngology and Head and Neck Surgery, Diakonessenhuis, Bosboomstraat 1, 3582 KE Utrecht, The Netherlands; 2 Department of Nuclear Medicine, Diakonessenhuis, Utrecht, The Netherlands

**Keywords:** 18F-NaF PET/CT, Bioactive glass, S53P4, Obliteration, Chronic otitis media

## Abstract

**Purpose:**

Bioactive glass has been successfully used for surgical treatment of chronic infections in bone and bone cavities. Besides infection control, new bone formation is induced by the bioactive glass which is considered to have osteoconductive properties. Evaluation of postsurgical changes after bone graft surgery is generally performed with conventional radiographs or CT/MR imaging, but 18F-NaF PET/CT might be more suitable since it has a high and rapid bone uptake, accompanied by a fast blood clearance leading to a high bone to background ratio.

**Case:**

Obliteration with S53P4 bioactive glass of the mastoid and middle ear was performed in a patient suffering from chronic otitis media. Control of the chronic otitis media was achieved, and follow-up imaging after 3 years with 18F-NaF PET/CT showed increased uptake in the obliterated cavity indicating new bone formation.

**Conclusion:**

18F-NaF PET/CT is able to detect new bone formation after obliteration of the mastoid with S53P4 bioactive glass.

## Main text

The use of bioactive glass for treatment of bone infections and infected bone cavities is a novel and promising approach for surgical control of chronic infections. It has been proven successful in surgical treatment of cavitary bone defects in chronic osteomyelitis (Lindfors et al. 2010a; Romanò et al. 2014; Ferrando et al. 2017). Furthermore, in chronic otitis media, control of infection was achieved by obliteration with bioactive glass of the mastoid cavity, respectively (Silvola 2012; Vos et al. 2017).

Bioactive glasses are composed of silica and calcium phosphate and have two main features: bacterial growth inhibition and stimulation of bone formation. The stimulation of bone formation is based on two characteristics of the bioactive glass: osteostimulation and osteoconduction. Osteostimulation refers to osteoblast cell recruitment and differentiation as well as osteoblast activation to produce new bone in a bony environment. The osteostimulative capacity of bioactive glass is attributed to a silica gel layer which is formed on the granules after obliteration, attracting calcium phosphate. Calcium phosphate consequently crystallizes into hydroxyapatite, similar to the mineral component of bone. Osteoconduction by the bioactive glass occurs by providing a latticework or scaffolding for bone to grow along in vivo. Other synthetic bone graft substitutes like demineralized bone matrix or ceramics like hydroxyapatite or tricalcium phosphate also have these osteostimulative and osteoconductive properties. Autogenous cancellous or cortical bone grafts have an extra feature to stimulate bone formation, i.e., osteoinduction. This is the process through which pluripotent mesenchymal cells from surrounding tissue are stimulated by growth factors within the graft to migrate and differentiate into osteoblasts. Assessing bone grafts in patients using imaging, either conventional radiographs, CT, MR, or bone scintigraphy, SPECT, and PET, thus requires knowledge about these phenomena (Beaman et al. 2006).

The golden standard for evaluation of bone formation is to take specimen for histology after surgery. However, this is in almost all cases not possible in humans, and therefore, evaluation of bone substitute incorporation in the host bone is generally performed with conventional radiographs or CT/MR imaging. With these imaging modalities, the cortical thickness can be described but active bone formation with these imaging modalities is not possible. 18F-NaF-PET/CT might be more suitable for this purpose. This very sensitive modality for detecting increased bone activity or remodeling uses 18F-NaF as a bone-seeking agent. The uptake of 18F-NaF reflects blood flow as well as bone remodeling (mineralization and bone formation); it has a high and rapid bone uptake, accompanied by a fast blood clearance leading to a high bone to background ratio. 18F-NaF-PET/CT has an improved specificity because the CT component of the hybrid system allows morphologic characterization of the functional lesion. Its use for assessment of bone metabolism in malignant skeletal diseases is extensively evaluated, but the technique has also been described for evaluation of postsurgical changes after bone graft surgery (Sörensen et al. 2003; Ullmark et al. 2007) and other benign bone diseases (Ovadia et al. 2007). Imaging of new, active bone formation with 18F-NaF-PET/CT after allograft obliteration in the mastoid has not been evaluated to date. Our objective was to visualize in vivo new bone formation induced by bioactive glass with 18F-NaF-PET/CT.

## Case

A 67-year-old patient frequently visited the department of otorhinolaryngology because of pain and discharge of the right ear caused by a chronic suppurative otitis media. Topical and systemic treatment with antibiotics, as well as surgical treatment by means of a cortical mastoidectomy, did not control the disease, and one episode of otitis media resulted in inner ear damage and finally profound deafness. In order to control the chronic ear infection, it was decided to perform a subtotal petrosectomy with obliteration of the cavity. This is a last resort surgical procedure and includes a total exenteration of all air cells in the temporal bone with preservation of vital structures such as the carotid artery, facial nerve, cochlea, and labyrinth. In most cases, the cavity is obliterated with soft tissue like abdominal fat. We decided to use bioactive glass S53P4 (BAG-S53P4, BonAlive Biomaterials Ltd., Turku, Finland) as obliteration material because of its antibacterial properties and its ability to control chronic suppurative otitis media in a series of patients who underwent mastoidectomy and obliteration with bioactive glass (Vos et al. 2017). In September 2015, the subtotal petrosectomy was performed with S53P4 bioactive glass obliteration and went uncomplicated.

**Fig. 1:**
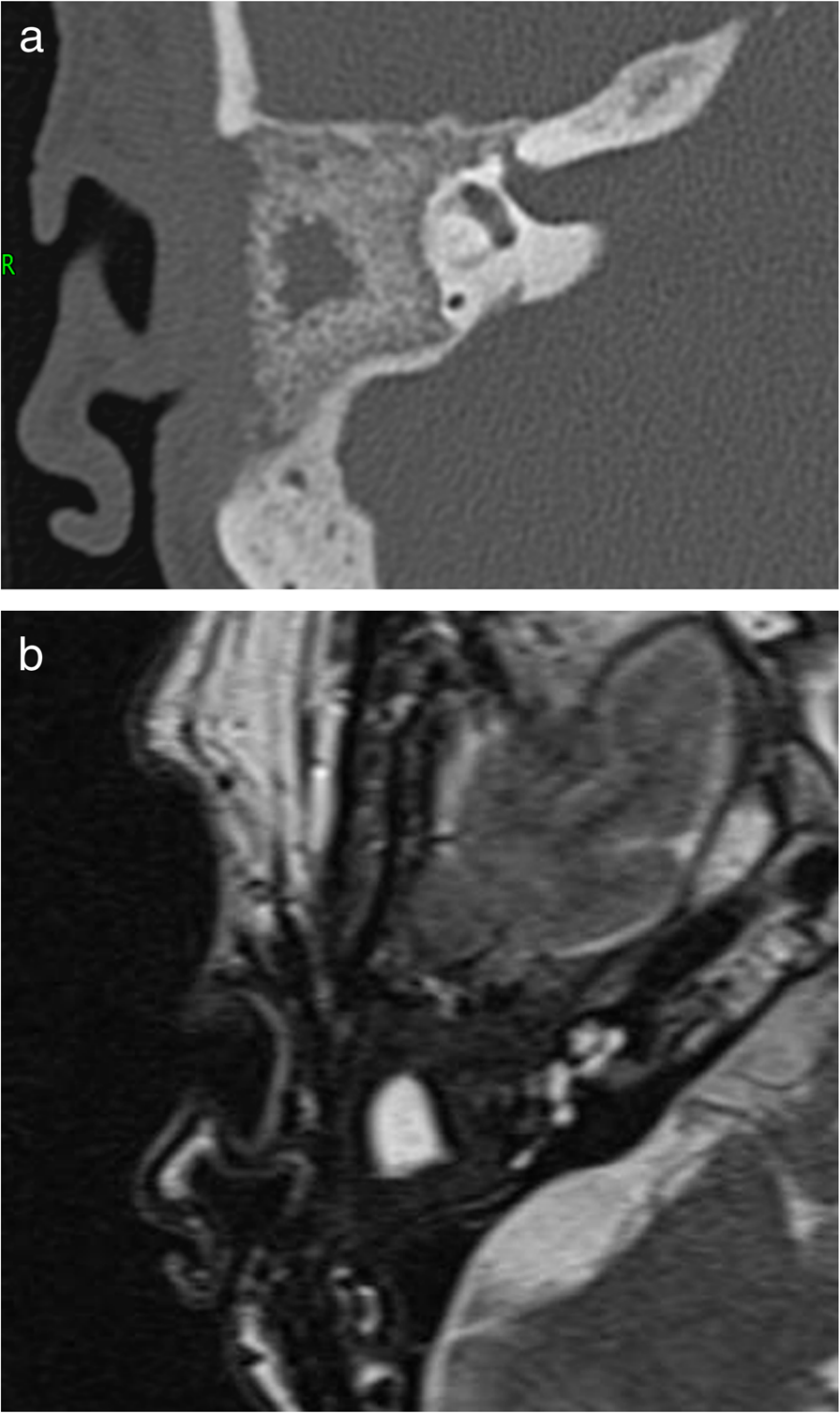
**a** Axial CT of the right mastoid showing a homogenous distribution of the bioactive glass S53P4 in the cavity of the middle ear and mastoid with a central hypodense area considered to represent seroma or fluid. **b** T2-weighted axial MRI of the same level

**Fig. 2:**
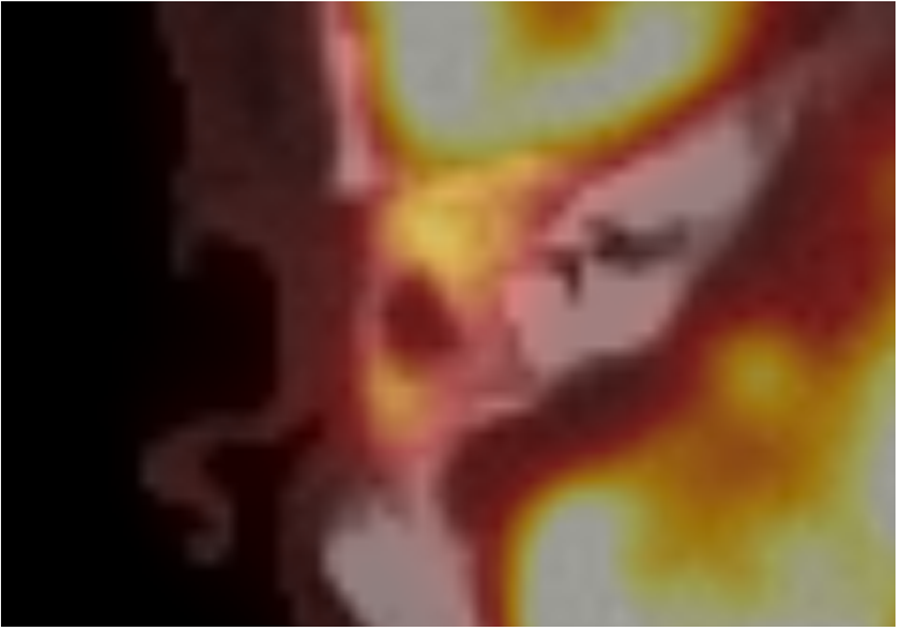
Axial 18F-FDG-PET/CT revealing increased uptake of the obliterated area, without uptake in the centrally located fluid area. The FDG uptake was considered too low for infection, but more likely a visualization of metabolic activity in the obliterated mastoid and middle ear cavity

**Fig. 3:**
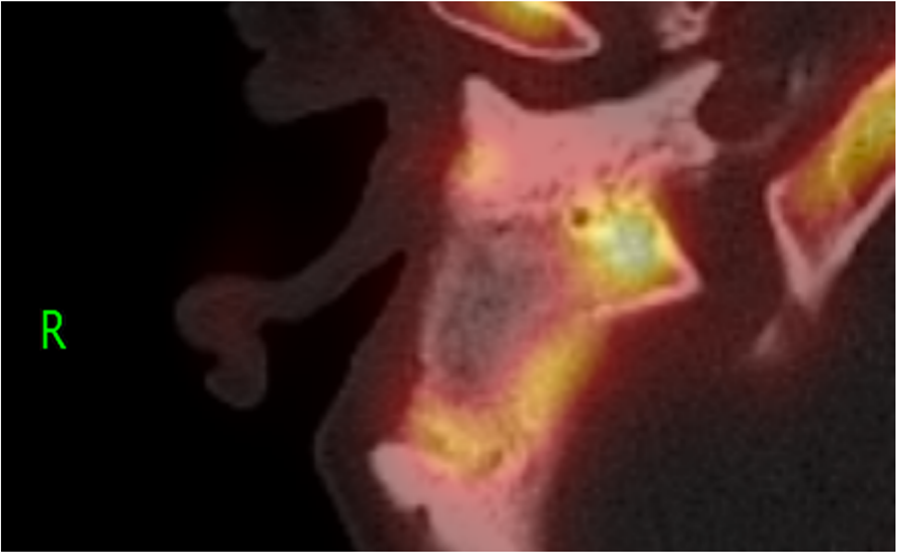
18F-NaF PET/CT showing increased uptake of 18F-NaF at the edges of the obliterated cavity. It was considered as osteoblast activity induced by bioactive glass S53P4 indicating formation of new bone

Control of the chronic suppurative otitis media was achieved, and follow-up imaging after 1 year with CT and MR showed a homogeneous distribution of the S53P4 bioactive glass in the surgical cavity, with an area of hypodensity in the center, matching with fluid or seroma (Fig. 1). The patient noticed, intermittently, in episodes of several days, a feeling of pressure in the temporal region of the right side, without any signs of infection. His complaints could not be explained by the CT and MR imaging. Therefore, 18F-fluorodeoxyglucose (FDG)-PET/CT was performed showing FDG uptake on the border of the cavity without FDG uptake in the central area of hypodensity (Fig. 2). The FDG uptake was considered too low for infection, but more likely a visualization of (reactive) metabolic activity in the obliterated mastoid and middle ear cavity. However, distinction between scar tissue, foreign body reaction, and bone formation could not be made. In order to attempt to visualize bone formation induced by the bioactive glass, 18F-NaF PET/CT was performed showing increased uptake of 18F-NaF at the edges of the obliterated cavity. It was considered as osteoblast activity induced by bioactive glass 3 years after the subtotal petrosectomy with obliteration of the mastoid and middle ear (Fig. 3).

## Discussion

The 18F-NaF uptake in the obliterated mastoid in our patient was considered to represent active bone formation 3 years after surgery where the mastoid was obliterated with bioactive glass S53P4. 18F-NaF PET/CT has been studied in benign bone disorders like in the evaluation of bone fractures or Paget’s disease and offers increased specificity, sensitivity, and diagnostic accuracy as compared to conventional bone scintigraphy and SPECT/CT (Li et al. 2012). Although Tc99m-diphosponate is the most commonly used bone tracer in conventional bone scintigraphy and the use of SPECT/CT offers the possibility of more detailed evaluation of postoperative changes after bone surgery, the accuracy of 18F-NaF PET/CT is superior, also if compared to FDG-PET/CT. 18F-NaF PET/CT is a superior technique, as compared to FDG-PET/CT, for assessment of bone formation and makes distinction with (reactive) metabolic activity (Hsu et al. 2007). The uptake mechanism of 18F-NaF in the bone includes the exchange of 18F ions with hydroxyl ions (OH^−^) on the surface of the hydroxyapatite to form fluoroapatite, and since it is accompanied by a fast blood clearance, a high bone to background ratio is achieved with this imaging agent. Uptake is higher in new bone because of a higher availability of binding sides, and therefore, nearly all causes of increased new bone formation have increased ^18^F localization. Visualization will depend on several factors, including blood flow and the amount of new bone formation. Processes that result in minimal osteoblastic activity may therefore not be detected.

**Fig. 4:**
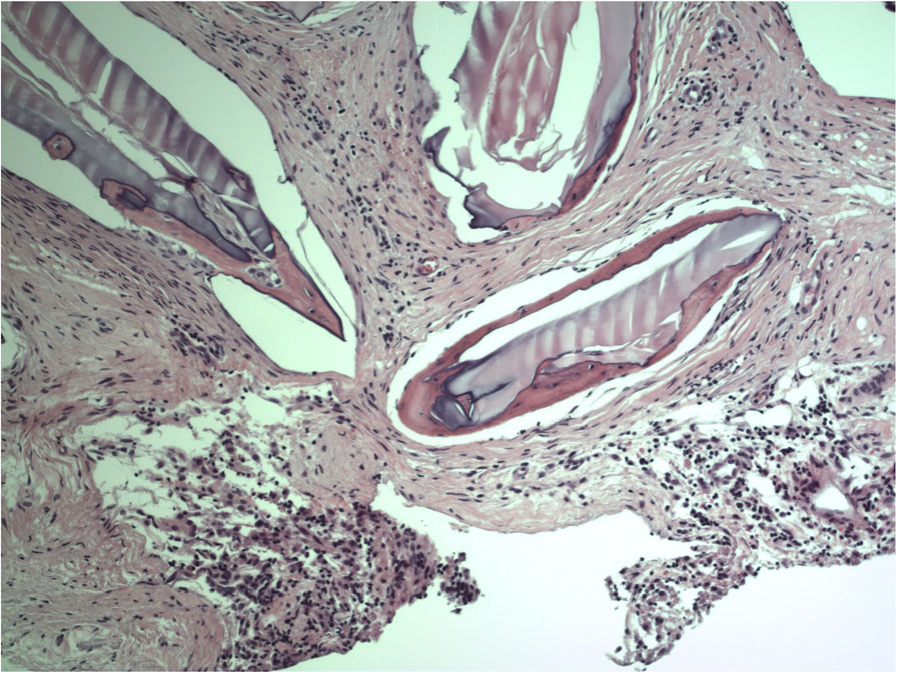
Histological specimen taken from an obliterated mastoid in a patient who underwent revision surgery for cholesteatoma showing osteoblast ingrowth between S53P4 glass granules indicating formation of new bone

Bioactive glass S53P4 is a synthetic biomaterial for treatment of bone infections or defects and infected bone cavities. Besides its ability to inhibit bacterial growth, it stimulates bone formation. The concept of osteostimulation by bioactive glass S53P4 has been proven in in vitro studies. Long-term follow-up of patients in which bioactive glass was used as bone graft subsitute in benign bone tumor surgery showed bone remodeling using x-ray and CT or MR imaging (Lindfors et al. 2010b). In a histological specimen taken of a mastoid obliterated with bioactive glass S53P4 in another patient who underwent revision surgery in our department for cholesteatoma, we found osteoblast ingrowth between the S53P4 glass granules indicating formation of new bone (Fig. 4).

Uptake of bone-seeking tracers is influenced by the synthetic bone substitute itself as well as the changes over time after surgery with insertion of grafts. Depending on local blood flow, tissue composition, dissolving of the synthetic bone substitute, and osteoblastic activity, variation in tracer uptake can occur. This can even lead to misleading findings as was described in a case with intense Tc99m-hydroxymethylene diphosphonate uptake 9 years after osteotomy and insertion of synthetic hydroxyapatite and tricalcium phosphate bone grafts (Tabouret-Viaud et al. 2014). Bioactive glass dissolves completely over years, and this will influence the uptake of bone-seeking tracers. In our patient, the 18F-NaF PET/CT was made 3 years after surgery. To our knowledge, no studies describing variation in tracer uptake, like quantification indexes (SUV), as well as taking into account the number of years after surgery with obliteration using bioactive glass have been published to date. Further evaluation in an experimental setting or a larger series of patients in which bone formation after mastoid obliteration is assessed with 18F-NaF PET/CT is therefore necessary.

We conclude that evaluation of postsurgical changes or bone formation with 18F-NaF PET/CT is feasible and might be of clinical importance in the follow-up of patients after bone graft surgery.

## Conclusion

18F-NaF PET/CT is able to visualize new bone formation induced by bioactive glass S53P4.

## Data Availability

Not applicable.

## Abbreviations

18F: Fluorine-18
CT: Computed tomography
FDG: Fluorodeoxyglucose
MR: Magnetic resonance
NaF: Sodium fluoride
OH: Hydroxyl ions
PET: Positron emission tomography
S53P4: Bioactive glass-S53P4, BonAlive Biomaterials Ltd., Turku, Finland
SPECT: Single photon emission computed tomography
Tc99m: Technetium-99m
